# A Multiwell Electrochemical Biosensor for Real-Time Monitoring of the Behavioural Changes of Cells *in Vitro*

**DOI:** 10.3390/s100403732

**Published:** 2010-04-13

**Authors:** Daman J. Adlam, David E. Woolley

**Affiliations:** University Dept Medicine, University of Manchester, MRI, Oxford Rd, Manchester, M13 9WL, UK; E-Mail: davidewoolley@live.co.uk

**Keywords:** Oncoprobe, electrochemical, biosensor, HepG2, cycloheximide, camptothecin

## Abstract

We report the development of a multiwell biosensor for detecting changes in the electrochemical open circuit potential (OCP) generated by viable human cells *in vitro*. The instrument features eight culture wells; each containing three gold sensors around a common silver/silver chloride reference electrode, prepared using screen-printed conductive inks. The potential applications of the device were demonstrated by monitoring rheumatoid synovial fibroblasts (RSF) and HepG2 hepatocarcinoma cells in response to chemical and biological treatments. This technology could provide an alternative to conventional end-point assays used in the fields of chemotherapy, toxicology and drug discovery.

## 1. Introduction

We have previously reported the principles of an electrochemical biosensor, ‘Oncoprobe’, for passively recording the Electrochemical open circuit potential (OCP) of confluent cell monolayers on gold sensors *in vitro* and its application in anticancer drug testing [1–3]. The technology operates by monitoring the electrochemical potential generated at the microenvironment of the cell:sensor interface, measured relative to a reference electrode located in the bulk culture medium. Changes in cell behaviour, such as those induced by anticancer drugs, inflammatory cytokines or other agents were shown to modify the OCP of viable cells *in vitro*.

Whole-cell biosensors are currently receiving increased interest since they can provide real-time, non-invasive and label-free detection in applications such as drug discovery, toxicology and theranostics [4,5]. Conventional spectroscopy-based cell assays, such as MTT, designed to assess cell proliferation, metabolic changes or cytotoxicity are often reliant upon given end-points, and frequently involve destruction of the cells under analysis, as critically assessed in recent reports [6,7]. A major limitation of many conventional assays is that data is acquired at one specific time point, whereas many biological and cellular processes proceed over variable time courses in response to different extraneous stimuli/ligands. Biosensor technologies have been developed to overcome the limitations of these assays and several approaches have been taken to achieve this; including impedance [8], quartz crystal microbalance [9,10], optical [11], ion selective [12] and electrochemical methods [13] of detection. Here we report on developments on the original assay design, whereby the solid gold electrodes (sensors) and calomel reference electrodes previously employed have been replaced with a miniaturised disposable ceramic probe with screen-printed gold sensors and silver/silver chloride reference electrodes suitable for an eight-well assay device. We demonstrate the application of the technology using *in vitro* cell cultures of synovial fibroblasts and the human hepatocellular carcinoma cell line, HepG2.

## 2. Results and Discussion

### 2.1. Oncoprobe Equipment

**Figure 1.**
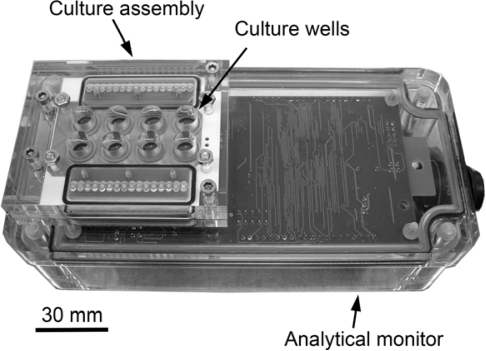
Oncoprobe instrument: Analytical monitor and 8-well cell culture assembly.

**Figure 2.**
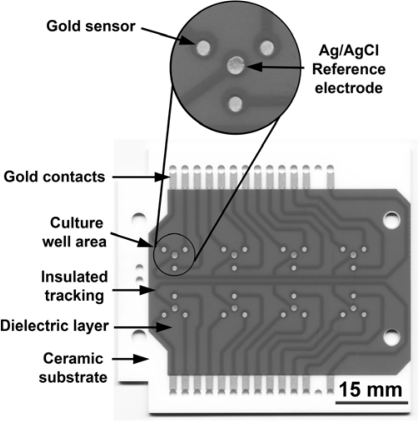
Disposable ceramic probe featuring gold sensors/tracking/contacts, reference electrode and insulating dielectric layer.

**Figure 3.**
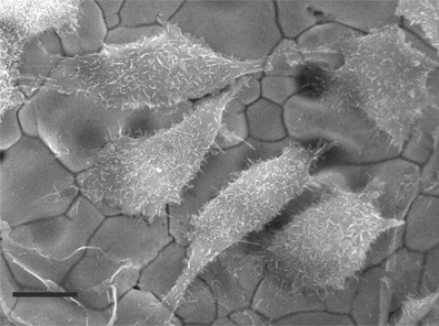
Scanning electron micrograph of human 8701-BC cells on a screen-printed, conductive gold electrode (sensor). Bar = 12 μm. (Courtesy of Prof. T.D. Allen, PICR, Manchester, UK).

Figure 1 shows the 8-well cell culture assembly connected to the analytical monitor unit. The disposable ceramic probe, which forms the base of the culture assembly, is illustrated in Figure 2. Biocompatibility and suitability of all the components of the bioassay device was tested, particularly the interaction of cells with the gold substratum. Demonstrated in Figure 3 by the adherence of human breast carcinoma cells (8701-BC) to the gold sensor as judged by scanning electron microscopy, 24 h after seeding; such attachment of viable cells being essential for the generation of an electrochemical signal (OCP). These cells have previously been shown to adhere to solid gold sensors [1] and successfully attached to the screen printed conductive ink gold sensors employed here.

The Oncoprobe instrument has developed through a number of key design features. Progression from an independent single well prototype unit to a disposable multiwell device; miniaturisation, which has allowed the number of cells and amount of media required per assay to be significantly reduced and the screen printing manufacturing process employed confers a high degree of reproducibility and reliability. Temperature and pH, factors we have previously shown to be important to the OCP signal obtained from sensors, have benefitted from greater stability due to operation within the controlled environment of a CO_2_ incubator [1]. In addition to the hardware developments, the polarity of the OCP displayed has been reversed from our earlier work [1,2], *i.e*., a more positive potential represents a more reducing state, whereas a more negative potential represents a more oxidising state. This enables us to attribute the changes in OCP observed definitively, hence a reduction in OCP now equates to a ‘loss’ of signal. Despite this change, the principal of the biosensor system remains the same; variations in the OCP of adherent cells on the gold sensors reflect changes in cell behaviour.

### 2.2. Electrochemical Monitoring of Cell Behaviour

Unlike conventional sensors, in whole cell biosensors such as the Oncoprobe system the cells form the active sensing layer. This provides the opportunity to not only assay cells from an individual against certain treatments, e.g., in chemosensitivity or drug resistance testing, but also for drug discovery or toxicology applications, whereby a target cell type or line could have candidate compounds screened against it.

**Figure 4.**
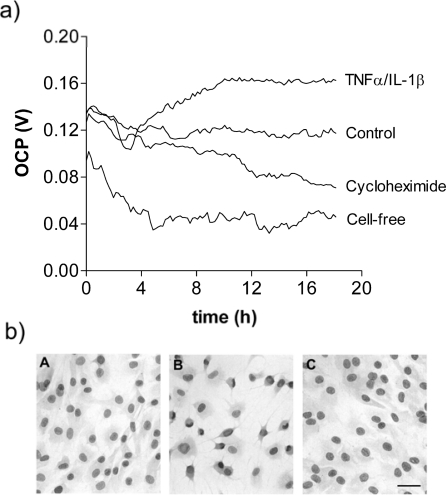
(a) Real-time monitoring of the OCP of rheumatoid synovial fibroblasts showing the effects of cycloheximide and TNFα/IL-1β (mean values from 6 sensors for each treatment). (b) Photomicrographs showing the morphology of control (A), TNFα/IL-1β (B) and cycloheximide (C) -treated cells after 18h. Bar = 20 μm.

Figure 4a shows the real-time monitoring of confluent layers of fibroblasts over 18h with and without treatments of cycloheximide or IL-1β/TNFα. Exposure to the latter is known to stimulate cellular activity and the OCP value is shown to be elevated over that of the control untreated cells. By contrast, cycloheximide (a known inhibitor of protein synthesis) is seen to suppress the OCP value of the cells. Thus, the electrochemical monitoring clearly discriminates between ‘activated’ and ‘compromised’ cells relative to the untreated controls. Confirmation that the treatments used in Figure 4a were non-toxic and cells remained adherent is demonstrated by the photomicrographs in Figure 4b; the cellular morphology observed for some cells exposed to TNFα/IL-1β showing a typical dendritic/stellate appearance (B in Figure 4b) compared to control and cycloheximide-treated cells.

**Figure 5.**
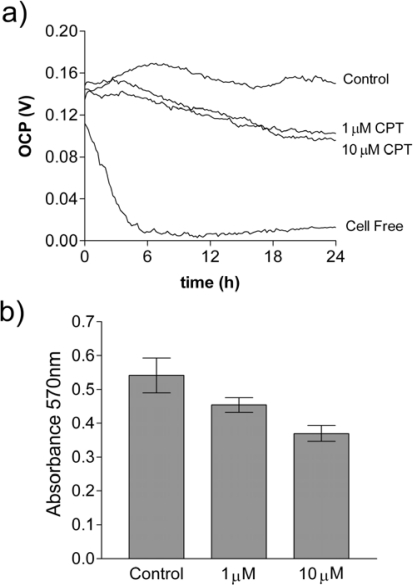
(a) Real-time electrochemical monitoring of the effect of camptothecin (CPT) on the OCP of HepG2 cells over 24 h (n = 6). (b) MTT assay data (absorbance ± SEM) from CPT treated HepG2 cells in parallel cultures after 24h (n = 6).

To demonstrate that this electrochemical technology is applicable to different adherent cell types, OCP values have been monitored in experiments involving a variety of cell lines. Figure 5a shows the OCP profile for the human hepatocyte cell line, HepG2, and its response to camptothecin, a cytotoxic quinoline alkaloid that inhibits DNA topoisomerase I and induces apoptosis [14]. For a comparative study, the same cell line and camptothecin concentrations were examined by the conventional MTT assay after 24 h (Figure 5b). Both assays demonstrate the effects of camptothecin exposure, but while the end-point MTT assay shows the effect of the compound after 24h, the real-time bioelectrochemical monitoring of Figure 5a shows behavioural changes throughout the time course; with adherent cells present beyond 24 h (data not shown).

In both the RSF and HepG2 electrochemical monitoring data (Figure 4a and Figure 5a) there is an initial period in which the signal takes time to settle. This is more pronounced in the cell-free sensors, suggesting that the presence of cells has a stabilising effect upon the signal. Analysis of the OCP generated by the confluent cell cultures prior to treatment (data not presented) showed that RSF generated a mean OCP of 112 mV (SD ± 7 mV) and HepG2 169 mV (SD ± 10 mV) whereas cell-free sensors produced 54 mV (SD ± 9 mV) and 6 mV (SD ± 17 mV) respectively. Therefore, cell-free sensors were not only more susceptible to variation but this was also dependent upon the culture media employed.

The multiwell Oncoprobe technology provides true real-time monitoring of the electrochemical signal. Therefore, quantitative data of the OCP observed can be defined over time, allowing a better understanding of the dynamic nature of cellular responses. The electrochemical reactions generating the OCP signal are yet to be conclusively defined but in our previous studies we have acknowledged the importance of redox and ionic changes at the cell:sensor interface, with dissolved oxygen concentration and pH values important factors [1,2]. Similarly, studies using scanning electrochemical microscopy (SECM) have also shown differences in the redox activity of normal and breast cancer cells [15]. Unlike impedance based biosensor technology [8,16,17] the flow of current in the Oncoprobe system is effectively negligible due to the high impedance employed. As such, the electrochemical monitoring of cell behaviour by this method is largely passive in comparison.

The experiments using RSF and HepG2 cells reported here illustrate the advantages of real-time electrochemical analysis, whereby the effects of various ligands may be analysed over time without the need to define specific end-points. For example, in Figure 4, differences in the OCP of cytokine, cycloheximide and control RSF cultures can be observed after just four hours from treatment. Similarly, in HepG2 cells treated with the cytotoxic, apoptosis inducing agent camptothecin (Figure 5) differences in the OCP are apparent less than six hours into the experiment.

The electrochemical OCP signal from confluent cultures of different cell lines have shown variations in the final plateaued value, which invites the question as to what processes contribute to the signal. Unlike RSF, which are restricted to confluent monolayers due to contact inhibition, cancer cell lines, although adherent to the gold sensors, may overgrow the single layer of cells, depending upon their proliferative rate. For this reason, the observations from parallel chamber slide cultures provide important information, not least an assessment of cell confluence and, if necessary, the fixation and staining of specific processes/proteins/morphology. Although the data presented is derived from adherent cell cultures that have reached confluence, earlier monitoring has the potential to provide information on cell attachment, spreading and proliferation rates.

## 3. Experimental Section

### 3.1. Oncoprobe Apparatus

The Oncoprobe biosensor unit is comprised of an eight well culture assembly featuring a re-usable acrylic culture vessel with disposable ceramic probe attached to an analytical monitor. The disposable ceramic probe is sandwiched into the culture assembly with silicon o-ring seals and secured with stainless steel M3 bolts and screws. Each well contains three 1mm diameter gold sensors around a central 1mm diameter silver/silver chloride reference electrode manufactured using screen printed conductive inks (DuPont, Bristol, UK). Electrodes are then connected by gold tracking to the edges with a dielectric insulating layer printed above. The gold and dielectric layers were screen printed and each fired at 850 °C for 60 minutes, reference electrodes were printed over gold vias and fired at 130 °C for 20 minutes. Gold-plated spring-loaded contacts (Coda Systems, Halstead, UK) interface the probe and analytical monitor via a bridging printed circuit board (PCB) embedded into the culture assembly. Silicon o-rings maintain an atmospheric seal between the analytical monitor and cell culture assembly. The analytical monitor was designed and manufactured for Oncoprobe Ltd. by Uniscan Instruments Ltd, Buxton, UK. Electrochemical open circuit potentials (OCP) generated at the cell:sensor interface are measured and processed by the analytical monitor, then displayed in real-time via proprietary software on a personal computer. The high impedance measurement system results in a negligible current flow within the system of less than 10^−15^ amps cm^−2^. A sampling frequency of once every ten minutes was employed for all experiments. The Oncoprobe biosensor unit operates within an incubator at 37 °C, 5%CO_2_ in humidified air.

### 3.2. Cell Cultures

Human rheumatoid synovial fibroblasts (RSF) and 8701-BC breast carcinoma cells were cultured in Dulbecco’s modified eagles medium (DMEM) supplemented with 10% foetal bovine serum (FBS) and antibiotic/antimycotic, as previously described [1]. Human hepatocyte carcinoma (HepG2) cells (ECACC, Porton Down, UK) were maintained in Eagles’ minimal essential medium (EMEM) supplemented with 10% FBS. All three cell types were harvested using 0.2% trypsin solution (all Gibco, Paisley, UK), centrifuged and resuspended at the desired density after a haemocytometer cell count. Sterile Oncoprobes were assembled and pre-incubated with culture medium:10% FBS for 2 hours before seeding cells.

### 3.3. Scanning Electron Microscopy

8701-BC cells were cultured on sections of the conductive gold ink coated ceramic probe. Following incubation overnight, samples were pre-fixed in 3% glutaraldehyde in Sorenson’s buffer (pH 7.4) for 1 hour, rinsed in buffer then post-fixed in 1% osmium tetroxide for 1 hour. After dehydration in ethanol and critical point drying by liquid CO_2_, 6 nm of chromium was sputter coated onto each section of ceramic probe. Specimens were viewed in a Topcon/ABT/ISI/DS130Fin lens, scanning electron microscope, as previously described [2].

### 3.4. Rheumatoid Synovial Fibroblasts (RSF)

RSF cells were seeded into six Oncoprobe wells at a density of 5 × 10^4^ cells well^−1^ in a 300 μL volume, the remaining two wells received medium alone. Eight-well Lab-Tek II chamber slides (VWR Intl, UK) were prepared in parallel, with each well receiving 8 × 10^4^ cells well^−1^ in a 475 μL volume. Cultures were incubated overnight, adherence and confluence were then evaluated in the chamber slides before aspirating cell culture wells and replenishing with medium containing 30 μM Cycloheximide (Sigma, Poole, UK), 0.5 ng mL^−1^ interleukin-1 beta (IL-1β) together with 20 ng mL^−1^ tumour necrosis factor alpha (TNFα) (R&D Systems, Abingdon, UK) in DMEM containing 2% FBS. Untreated RSF and cell-free controls received medium:FBS alone. Oncoprobe cultures were monitored for 18 h. Chamber slides containing treated and untreated control cells were fixed with 70% ethanol after 18 h, air-dried and stained with Harris’ Haematoxylin (Sigma, Poole, UK). Slides were viewed and photomicrographs taken with a Motic DM-BA300 digital photomicroscope system.

### 3.5. HepG2 Toxicity Assay

HepG2 cells in EMEM:10% FBS were seeded onto Oncoprobes at a density of 5 × 10^5^ cells well^−1^; cell-free control wells received media alone. Cells were also seeded into sterile 96-well plates (VWR, UK) at a density of 2 × 10^5^ cells well^−1^ for the 3-[4,5-Dimethylthiazol-2-yl]-2,5-diphenyltetrazolium bromide (MTT) based assay [18]. After incubating overnight, the wells were aspirated and treated with camptothecin (1–10 μM) in EMEM containing 2% FBS; HepG2 and cell-free controls received medium alone. Drug-induced effects were then monitored electrochemically for 24 h using the Oncoprobe system. The 96-well plates were assayed after 24h using an MTT based *in vitro* toxicology assay kit (Sigma, Poole, UK) as per the manufacturers’ instructions. Briefly, MTT solution was added to the cultures to a final concentration of 0.5 mg mL^−1^ and incubated for 2 hours. The resulting formazan was then dissolved by adding an equal volume of 10% Triton X-100, 0.1N HCl in anhydrous isopropanol. Absorbance values were measured spectrophotometrically using a Dynex MRX II plate reader at 570 nm with a 690 nm background measurement.

## 4. Conclusions

This study has shown that the multiwell electrochemical monitoring technology demonstrated here can detect changes in the open circuit potential generated by cells in response to various factors; activation via cytokines, inhibition of protein synthesis and induction of apoptosis all cause significant modification in the OCP generated by the cells. As such, the system has shown potential applications in research, toxicology and theranostics. While the developments described in this publication have enhanced the potential of the technology in such fields, we recognise that further research and development is required to identify the mechanisms by which the OCP is generated and fully validate its efficacy.

## References

[1] Woolley D.E., Tetlow L.C., Adlam D.J., Gearey D., Eden R.D. (2002). Electrochemical monitoring of cell behaviour *in vitro*: a new technology. Biotechnol. Bioeng.

[2] Woolley D.E., Tetlow L.C., Adlam D.J., Gearey D., Eden R.D., Ward T.H., Allen T.D. (2002). Electrochemical monitoring of anticancer compounds on the human ovarian carcinoma cell line A2780 and its adriamycin- and cisplatin-resistant variants. Exp. Cell Res.

[3] Adlam D.J., Dabbous M.K., Woolley D.E. (2008). Electrochemical monitoring of rat mammary adenocarcinoma cells: an *in vitro* assay for anticancer drug selection. Assay Drug Dev. Technol.

[4] Warner S. (2004). Diagnostics plus therapy = theranostics. Scientist.

[5] Mestres P., Morguet A. (2009). The bionas technology for anticancer drug screening. Expert. Opin. Drug Discov.

[6] Miret S., De Groene E.M., Klaffke W. (2006). Comparison of *in vitro* assays of cellular toxicity in the human hepatic cell line HepG2. J. Biomol. Screen.

[7] Mueller H., Kassack M.U., Wiese M. (2004). Comparison of the usefulness of the MTT, ATP, and calcein assays to predict the potency of cytotoxic agents in various human cancer cell lines. J. Biomol. Screen.

[8] Solly K., Wang X., Xu X., Strulovici B., Zheng W. (2004). Application of real-time cell electronic sensing (RT-CES) technology to cell-based assays. Assay Drug. Dev. Technol.

[9] Braunhut S.J., McIntosh D., Vorotnikova E., Zhou T., Marx K.A. (2005). Detection of apoptosis and drug resistance of human breast cancer cells to taxane treatments using quartz crystal microbalance biosensor technology. Assay Drug. Dev. Technol.

[10] Jia X., Tan L., Me Q.J., Zhang Y.Y., Yao S.Z. (2008). Quartz crystal microbalance and electrochemical cytosensing on a chitosan/multiwalled carbon nanotubes/Au electrode. Sensor. Actuator. B-Chem.

[11] Fang Y. (2006). Label-free cell-based assays with optical biosensors in drug discovery. Assay Drug Dev. Technol.

[12] Ghosh G., Mehta I., Comette A.L., Anderson K.W. (2008). Measuring permeability with a whole cell-based biosensor as an alternate assay for angiogenesis: Comparison with common *in vitro* assays. Biosens. Bioelectron.

[13] Torisawa Y.S., Kaya T., Takii Y., Oyamatsu D., Nishizawa M., Matsue T. (2003). Scanning electrochemical microscopy-based drug sensitivity test for a cell culture integrated in silicon microstructures. Anal. Chem.

[14] Liu E.H., Qi L.W., Wu Q., Peng Y.B., Li P. (2009). Anticancer agents derived from natural products. Mini-Rev. Med. Chem.

[15] Rotenberg S.A., Mirkin M.V. (2004). Scanning electrochemical microscopy: detection of human breast cancer cells by redox environment. J. Mammary Gland Biol. Neoplasi.

[16] Keese C.R., Bhawe K., Wegener J., Giaever I. (2002). Real-time impedance assay to follow the invasive activities of metastatic cells in culture. Biotechniques.

[17] Mitra P., Keese C.R., Giaever I. (1991). Electric measurements can be used to monitor the attachment and spreading of cells in tissue-culture. Biotechniques.

[18] Mosmann T. (1983). Rapid colorimetric assay for cellular growth and survival—application to proliferation and cyto-toxicity assays. J. Immunol. Method.

